# Physiological and Biochemical Responses of *Ulva prolifera* and *Ulva linza* to Cadmium Stress

**DOI:** 10.1155/2013/289537

**Published:** 2013-03-06

**Authors:** He-ping Jiang, Bing-bing Gao, Wen-hui Li, Ming Zhu, Chun-fang Zheng, Qing-song Zheng, Chang-hai Wang

**Affiliations:** ^1^College of Natural Resources and Environmental Science, Key Laboratory of Marine Biology, Nanjing Agricultural University, Nanjing, Jiangsu 210095, China; ^2^Zhejiang Mariculture Research Institute, Zhejiang Key Laboratory of Exploitation and Preservation of Coastal Bio-Resource, Wenzhou, Zhejiang 325005, China; ^3^College of Oceanography, Huaihai Institute of Technology, Lianyungang, Jiangsu 222005, China

## Abstract

Responses of *Ulva prolifera* and *Ulva linza* to Cd^2+^ stress were studied. We found that the relative growth rate (RGR), Fv/Fm, and actual photochemical efficiency of PSII (Yield) of two *Ulva*species were decreased under Cd^2+^ treatments, and these reductions were greater in *U. prolifera* than in *U. linza*. *U. prolifera* accumulated more cadmium than *U. linza* under Cd^2+^ stress. While *U. linza* showed positive osmotic adjustment ability (OAA) at a wider Cd^2+^ range than *U. prolifera*. *U. linza* had greater contents of N, P, Na^+^, K^+^, and amino acids than *U. prolifera*. A range of parameters (concentrations of cadmium, Ca^2+^, N, P, K^+^, Cl^−^, free amino acids (FAAs), proline, organic acids and soluble protein, Fv/Fm, Yield, OAA, and K^+^/Na^+^) could be used to evaluate cadmium resistance in *Ulva* by correlation analysis. In accordance with the order of the absolute values of correlation coefficient, contents of Cd^2+^ and K^+^, Yield, proline content, Fv/Fm, FAA content, and OAA value of *Ulva* were more highly related to their adaptation to Cd^2+^ than the other eight indices. Thus, *U. linza* has a better adaptation to Cd^2+^ than *U. prolifera*, which was due mainly to higher nutrient content and stronger OAA and photosynthesis in *U. linza*.

## 1. Introduction

Heavy metal contamination is an environmental problem in the margin sea [1]. As the economy in Asian countries continues to grow, the release of heavy metals and other contaminants has increased noticeably [2, 3]. Due to their acute toxicity, cadmium (Cd), lead, and mercury are among the most hazardous metals to the environment and living things [4]. 

Cd, an oxophilic and sulfophilic element, forms complexes with various organic particles and thereby triggers a wide range of reactions that collectively put the aquatic ecosystems at risk. Cadmium also poses a serious threat to human health due to its accumulation in the food chain [5, 6]. It has been classified as group (I) a human carcinogen by the International Agency for Research on Cancer (IARC) [7]. Cadmium toxicity may be characterized by a variety of syndromes and effects, including renal dysfunction, hypertension, hepatic injury, lung damage, and teratogenic effects [8]. To remove Cd pollutants, various treatment technologies, such as precipitation, ion exchange, adsorption, and biosorption, have been employed [9]. Biosorption is one of the promising techniques for removal of heavy metals. Biosorption utilizes the ability of biological materials to accumulate heavy metals from waste streams by either metabolically mediated or purely physicochemical pathways of uptake [10]. Among the biological materials investigated for heavy metal removal, marine macroalgae have high uptake capacities for a number of heavy metal ions [11, 12].

Green algae species of Ulvaceae, especially the members of the green algal genus *Ulva*, have been considered as monitors of heavy metals in estuaries [13–15]. Numerous studies have shown that green macroalgae such as *Ulva lactuca* are able to absorb Cd. These studies mainly focused on metabolism-independent Cd accumulation [6], synthetic surfactants exerting impact on uptake of Cd [12], effect of pH, contact time, biomass dosage and temperature on the Cd uptake kinetics [2], and induced oxidative stress by Cd [7]. However, little information is available regarding physiological responses of different *Ulva* species to increased Cd^2+^ concentrations. 

In this study, *Ulva prolifera *and *Ulva linza *were studied for their responses to different Cd^2+^ concentrations. Their growth, chlorophyll fluorescence parameters, osmotic adjustment ability, and accumulation of inorganic ions and organic solutes were investigated in indoor seawater culture systems. The specific objective of this study was to determine if there was species variation in Cd^2+^ adaptation, and what were the major physiological parameters involved in the adaptation. 

## 2. Materials and Methods

### 2.1. The Seaweed Collection, Cultivation, and Cd^2+^ Treatment

Green algae were collected from the sea in Dafeng (*Ulva prolifera*) and Lianyungang (*Ulva linza*), Jiangsu province, China. Upon arrival in the laboratory, the seaweeds were washed with distilled water and then cultured in 250 mL flasks containing 200 mL of sterilized artificial seawater (33.33 psu, pH 8.0) enriched with VSE medium [16] for 5 d. The composition of artificial seawater was (g L^−1^) HCO_3_^−^ 0.25, SO_4_^2−^ 3.84, Cl^−^ 17.45, Ca^2+^ 0.76, Mg^2+^ 1.00, K^+^ 0.57, and Na^+^ 9.46. The composition of VSE nutrient solution was (mg L^−1^) NaNO_3_ 42.50, Na_2_HPO_4_·12H_2_O 10.75, FeSO_4_·7H_2_O 0.28, MnCl_2_·4H_2_O 0.02, Na_2_EDTA·2H_2_O 3.72, vitamin B_1_ 0.20, Biotin 0.001, and vitamin B_12_ 0.001. After 5 d acclimation, healthy samples (0.5 g fresh weight) were cultured in 250 mL flasks with 200 mL medium as described earlier. CdCl_2_ was added to each flask at the following concentrations: 0, 5, 10, 20, 40, 80, or 120 *μ*mol L^−1^. After 7 d treatment, *U. prolifera* and *U. linza* were harvested and analyzed for selected parameters as described later. All experiments were performed in three replicates. During the preculture and the treatment, seaweeds were grown in a GXZ intelligent light incubator at temperature of 20 ± 1°C, light intensity of 50 *μ*mol m^−2^ s^−1^, and photoperiod of 12/12 h. The culture medium was altered every other day. 

### 2.2. Measurement of Relative Growth Rate (RGR)

Fresh weight was determined by weighing the algae after blotting by absorbent paper. RGR was calculated according to the formula RGR (% d^−1^) = [ln⁡(*M*_*t*_/*M*_0_)/*t*] × 100%, where *M*_0_ and *M*_*t*_ are the fresh weights (g) at days 0 and 7, respectively [17]. 

### 2.3. Measurement of Osmotic Adjustment Ability (OAA)

Saturated osmotic potential was measured by the freezing-point depression principle. Seaweeds were placed in double-distilled water for 8 h and then rinsed 5 times with double-distilled water. After blotting dry with absorbent paper, seaweeds were dipped into liquid nitrogen for 20 min. The frozen seaweeds were thawed in a syringe for 50 min, and the seaweed sap was then collected by pressing the seaweed in the syringe [18]. The *π*_100_ was measured by using a fully automatic freezing-point osmometer (8P, Shanghai, China). OAA was calculated by the following equation:

(1) Δπ100=π100μ−π100s,

whereby *π*_100_^*μ*^ was the *π*_100_ of control seaweeds, and *π*_100_^s^ was the *π*_100_ of Cd^2+^-stressed seaweeds.

### 2.4. Measurements of Chlorophyll (Chl) and Carotenoid (Car) Contents

Determination of Chl and Car was carried out by the method of Häder et al. [19]. Weighed 0.1 g fresh seaweeds were cut with scissors and extracted with 95% (v/v) ethanol (10 mL) in the dark for 24 h. The absorbance of pigment extract was measured at wavelengths of 470, 649, and 665 nm with a spectrophotometer. From the measured absorbance, concentrations of Chl a, Chl b, and Car were calculated on a weight basis.

### 2.5. Determination of Chlorophyll Fluorescence Parameters

A PHYTO-PAM Phytoplankton Analyzer (PAM 2003, Walz, Effeltrich, Germany) was used to determine *in vivo* chlorophyll fluorescence from chlorophyll in photosystem II (PSII) using different experimental protocols [19]. Before determination, samples were adapted for 15 min in the total darkness to complete reoxidation of PSII electron acceptor molecules. The maximal photochemical efficiency of PSII (Fv/Fm) and the actual photochemical efficiency of PSII in the light (Yield) were then determined.

### 2.6. Measurement of Nitrogen (N) and Phosphorous (P) Concentrations

Dried samples were ground in a mortar and pestle. Total N in seaweed tissue was analyzed by an N gas analyzer using an induction furnace and thermal conductivity. Total P in seaweed tissue was quantitatively determined by Inductively Coupled Plasma Atomic Emission Spectrometry (ICP-AES, Optima 2100 DV, PerkinElmer, USA) following nitric acid/hydrogen peroxide microwave digestion. The total amounts of N and P in the seaweed tissue were calculated by multiplying N and P contents in tissue as a proportion of dry weight by the total dry weight of the sample [20]. 

### 2.7. Measurement of Inorganic Elements

After 7 d, seaweeds were harvested, washed, and oven-dried at 65°C for 3 d. A 50 mg sample was ashed in a muffle furnace. The ash was dissolved in 8 mL of HNO_3_ : HClO_4_ (3 : 1, v : v) and diluted to 50 mL with distilled water. The contents of Cd, Na, K, Ca, and Mg were determined by Inductively Coupled Plasma Atomic Emission Spectrometry (ICP-AES, Optima 2100 DV, PerkinElmer, USA) [21]. To determine Cl content, the ash was dissolved in 100 mL distilled water and analyzed by potentiometric titration with silver nitrate (AgNO_3_) [18]. Total nitrate was measured as described previously [22] with nitrate extracted from the tissue by boiling fresh seaweeds (20 mg) in distilled water (400 *μ*L) for 20 min. The nitrate concentrations in the samples were measured spectrophotometrically at 540 nm.

### 2.8. Measurement of Organic Solutes

Soluble sugars (SS) determination was carried out by the anthrone method [23]. Water extract of fresh seaweeds was added to 0.5 mL of 0.1 mol L^−1^ anthrone-ethyl acetate and 5 mL H_2_SO_4_. The mixture was heated at 100°C for 1 min, and its absorbance at 620 nm was read after cooling to room temperature. A calibration curve with sucrose was used as a standard. Soluble proteins (SPs) were measured by Coomassie Brilliant Blue G-250 staining [24]. Fresh seaweeds (0.5 g) were homogenized in 1 mL phosphate buffer (pH 7.0). The crude homogenate was centrifuged at 5,000 g for 10 min. An aliquot of 0.5 mL of freshly prepared trichloroacetic acid (TCA) was added and mixture centrifuged at 8.000 g for 15 min. The pellets were dissolved in 1 mL of 0.1 mol L^−1^ NaOH, and 5 mL of Bradford reagent was added. Absorbance was recorded at 595 nm using bovine serum albumin as a standard. Free amino acids (FAAs) were extracted and determined following the method of Zhou and Yu [23]. A total of 0.5 g fresh tissue was homogenized in 5 mL 10% (w/v) acetic acid, extracts were supplemented with 1 mL distilled water and 3 mL ninhydrin reagent, then boiled for 15 min and fast cooled, and the volume was made up to 5 mL with 60% (v/v) ethanol. Absorbance was read at 570 nm. The content of total free amino acids was calculated from a standard curve prepared using leucine. Proline (PRO) concentration was determined spectrophotometrically by adopting the ninhydrin method of Irigoyen et al. [25]. We first homogenized 300 mg fresh leaf samples in sulphosalicylic acid. To the extract, 2 mL each of ninhydrin and glacial acetic acid were added. The samples were heated at 100°C. The mixture was extracted with toluene, and the free toluene was quantified spectrophotometrically at 528 nm using L-proline as a standard. Organic acids (OAs) were extracted with boiling distilled water. The concentration of total OA was determined by 0.01 mmol L^−1^ NaOH titration method, with phenolphthalein as indicator [26].

### 2.9. Statistical Analyses

All experiments were performed in three replicates. The data are presented as the mean ± SD. Data were analyzed using SPSS statistical software. Significant differences between means were determined by Duncan's multiple range test. Unless otherwise stated, differences were considered statistically significant when *P* ≤ 0.05. Statistical analysis on two-way variance analysis (ANOVA), and correlation coefficient was performed using Microsoft Excel. 

## 3. Results

### 3.1. Effect of Cadmium Stress on RGR and OAA of *U. prolifera* and *U. linza*

**Figure 1:**
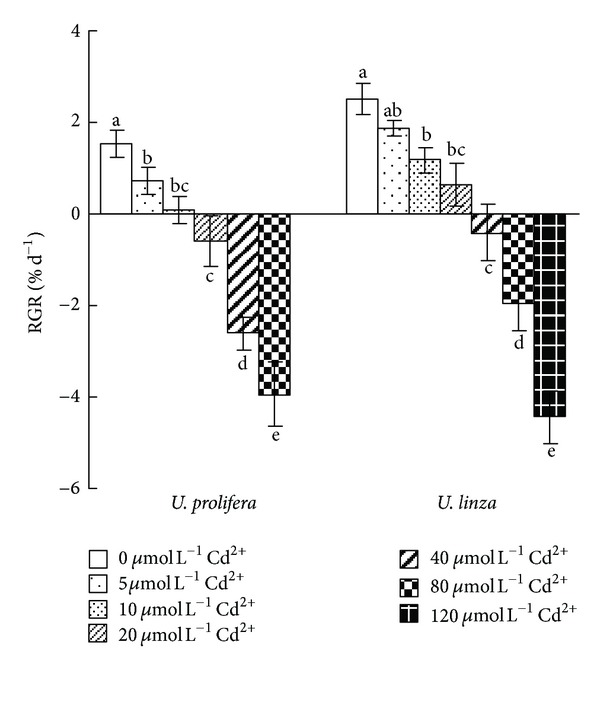
Effects of different concentrations of Cd^2+^ (0, 5, 10, 20, 40, 80, and 120**** *μ*mol L^−1^) on relative growth rate (RGR) in *U. prolifera *and* U. linza. *

Compared to the control, treatments with 5 *μ*mol L^−1^ Cd^2+^ for 7 d did not change RGR of *U. linza,* but significantly decreased RGR of *U. prolifera*. The RGR of both *Ulva* species was significantly decreased as Cd^2+^ concentration increased. After 7 d exposure to 10, 20, 40, 80; or 120 *μ*mol L^−1^ Cd^2+^, RGR of *U. linza* decreased by 53, 75, 116, 177, and 277%, respectively; *U. prolifera *decreased by 93, 139, 271, and 357%, respectively. *U. prolifera* died at 120 *μ*mol L^−1^ Cd^2+^ on day 7 (Figure 1). 

**Figure 2:**
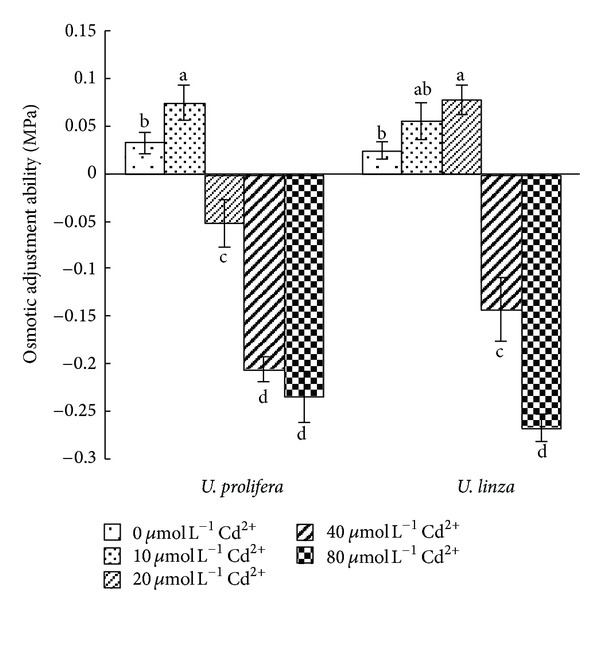
Effects of different concentrations of Cd^2+^ (5, 10, 20, 40, and 80**** *μ*mol** **L^−1^) on osmotic adjustment ability (OAA) of *U. prolifera *and* U. linza. *

The OAA of both species was enhanced by low Cd^2+^ concentration treatments. The enhancement occurred at 5 and 10 *μ*mol L^−1^ for *U. prolifera* and 5, 10 and 20 *μ*mol L^−1^ for *U. linza* (Figure 2). However, OAA was negative when *U. prolifera* was treated by 20, 40, and 80 *μ*mol L^−1^ Cd^2+^, and *U. linza* treated by 40 and 80 *μ*mol L^−1^ Cd^2+^ (Figure 2). 

### 3.2. Effect of Cadmium Stress on Cadmium Content in *U. prolifera* and *U. linza*

**Figure 3:**
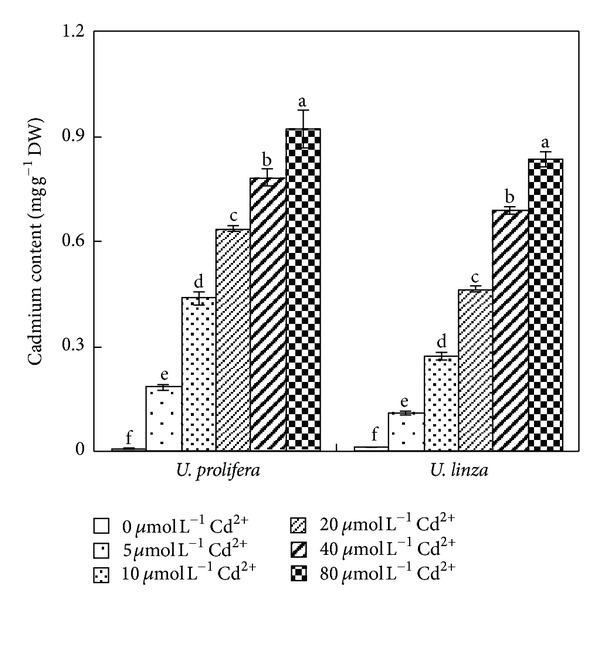
Effects of different concentrations of Cd^2+^ (0, 5, 10, 20, 40, and 80**** *μ*mol** **L^−1^) on cadmium concentration of *U. prolifera *and* U. linza. *

Cadmium contents in *U. prolifera *and* U. linza* increased as Cd^2+^ concentrations increased (Figure 3). At 5, 10, 20, 40, and 80 *μ*mol L^−1^Cd^2+^, Cd contents in *U. prolifera* was 32, 78, 114, 140, and 165 times of the Cd^2+^ = 0 treatment, respectively, and 10, 26, 44, 65, and 79 times of its control treatment in *U. linza*, respectively.

### 3.3. Effect of Cadmium Stress on Chl and Car Contents in *U. prolifera* and *U. linza*

**Figure 4:**
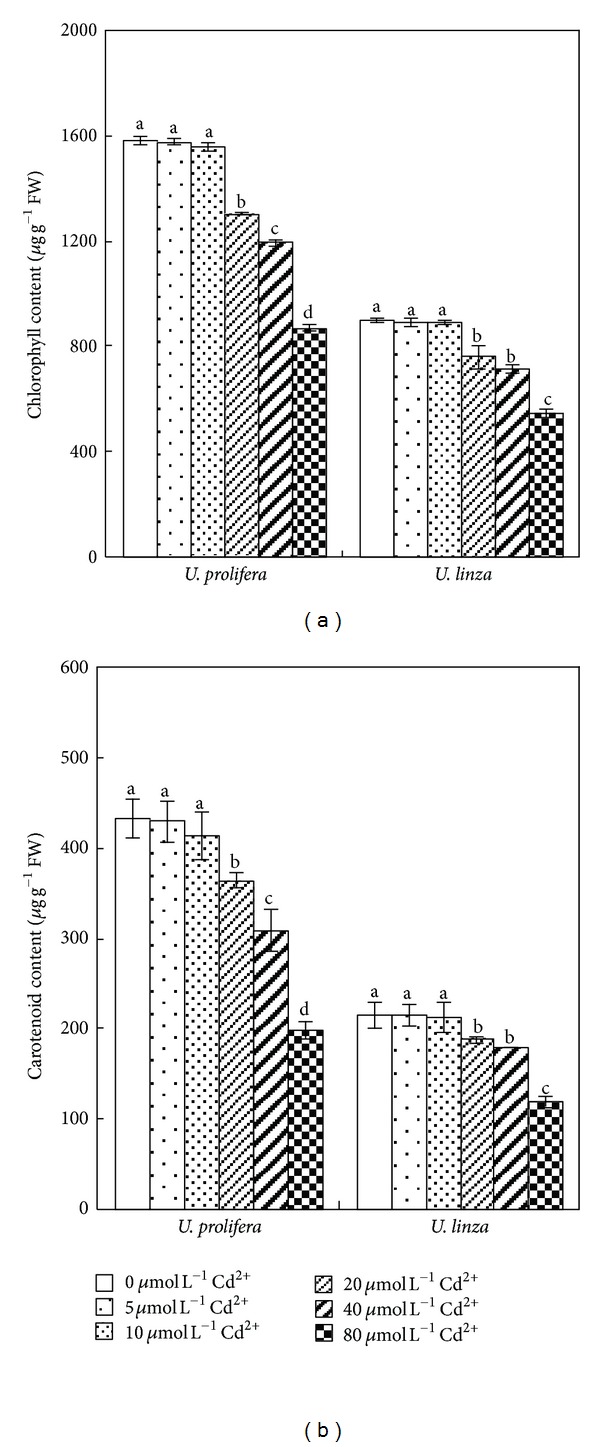
Effects of different concentrations of Cd^2+^ (0, 5, 10, 20, 40, and 80**** *μ*mol** **L^−1^) on chlorophyll content (a) and carotenoid content (b) in *U. prolifera and U. linza. *

Both Chl and Car contents decreased with the increased Cd^2+^ concentration. There was no significant change in Chl and Car when both species were treated by 5 and 10 *μ*mol L^−1^ Cd^2+^ for 7 d. However, significant declines in Chl and Car contents were observed when they were exposed to 20, 40, or 80 *μ*mol L^−1^ Cd^2+^. Compared to the control treatment, Chl contents decreased by 18, 25, and 45% at 20, 40, and 80 *μ*mol L^−1^ Cd^2+^ in *U. prolifera, *respectively; and the decreases were 16, 20, and 39% in *U. linza*, respectively (Figure 4(a)). The Car content declined by 16, 29 and 54% at 20, 40 and 80 *μ*mol L^−1^ Cd^2+^ in *U. prolifera*, respectively; and by 13, 16, and 44% in *U. linza*, respectively (Figure 4(b)).

### 3.4. Effect of Cadmium Stress on Chlorophyll Fluorescence Parameters of *U. prolifera* and *U. linza*

**Figure 5:**
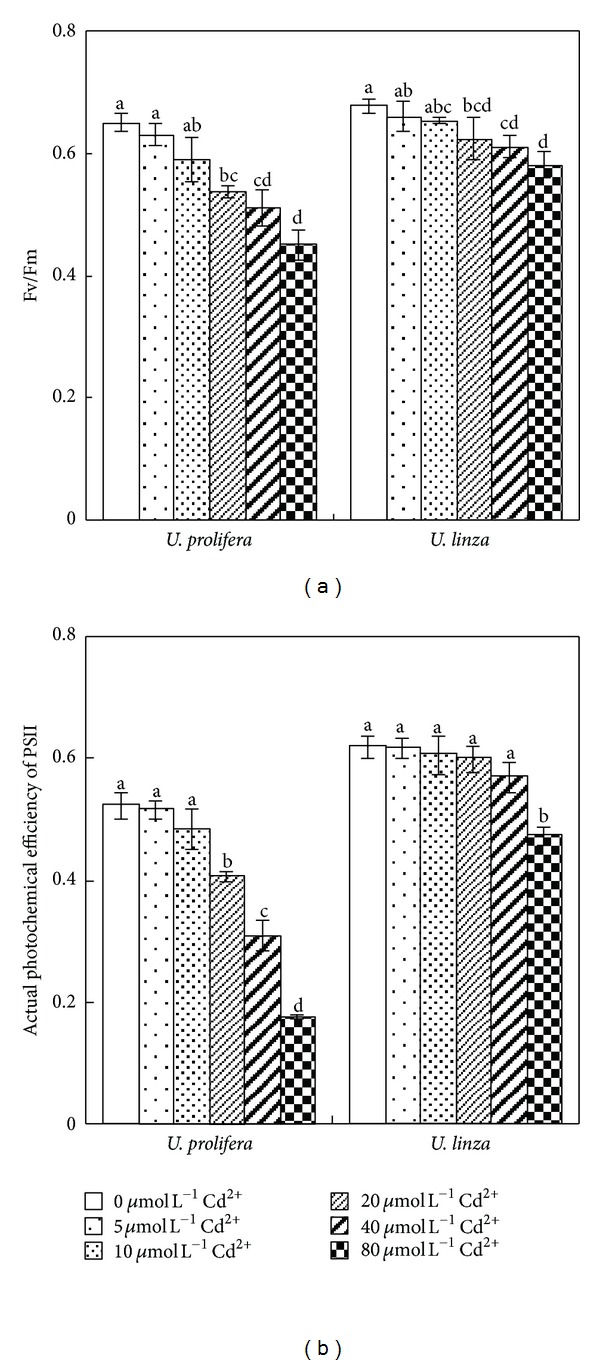
Effects of different concentrations of Cd^2+^ (0, 5, 10, 30, 40, and 80 *μ*mol L^−1^) on Fv/Fm (a) and Yield (actual photochemical efficiency of PSII) (b) of *U. prolifera* and *U. linza. *

Compared to the control treatment, Fv/Fm of *U. prolifera *and *U. linza* were not significantly affected by the treatments of 5 or 10 *μ*mol L^−1^ Cd^2+^. However, Fv/Fm of both *Ulva* species fell significantly when Cd^2+^ concentrations reached 20 *μ*mol L^−1^. In comparison with the control, Fv/Fm of *U. prolifera *decreased 17, 22, and 31% at 20, 40, and 80 *μ*mol L^−1^ Cd^2+^; whereas Fv/Fm of *U. linza* decreased 9, 10, and 15% after exposure to 20, 40, or 80 *μ*mol L^−1^ Cd^2+^, respectively (Figure 5(a)). For actual photochemical efficiency of PSII (Yield) of *U. prolifera*, there was an obvious decrease when Cd^2+^ concentrations rose from 20 to 80 *μ*mol L^−1^; whereas Yield of *U. linza* showed no significant decline until Cd^2+^ concentration was 80 *μ*mol L^−1^ (Figure 5(b)). 

### 3.5. Effect of Cadmium Stress on Contents of N and P in *U. prolifera* and *U. linza*

**Figure 6:**
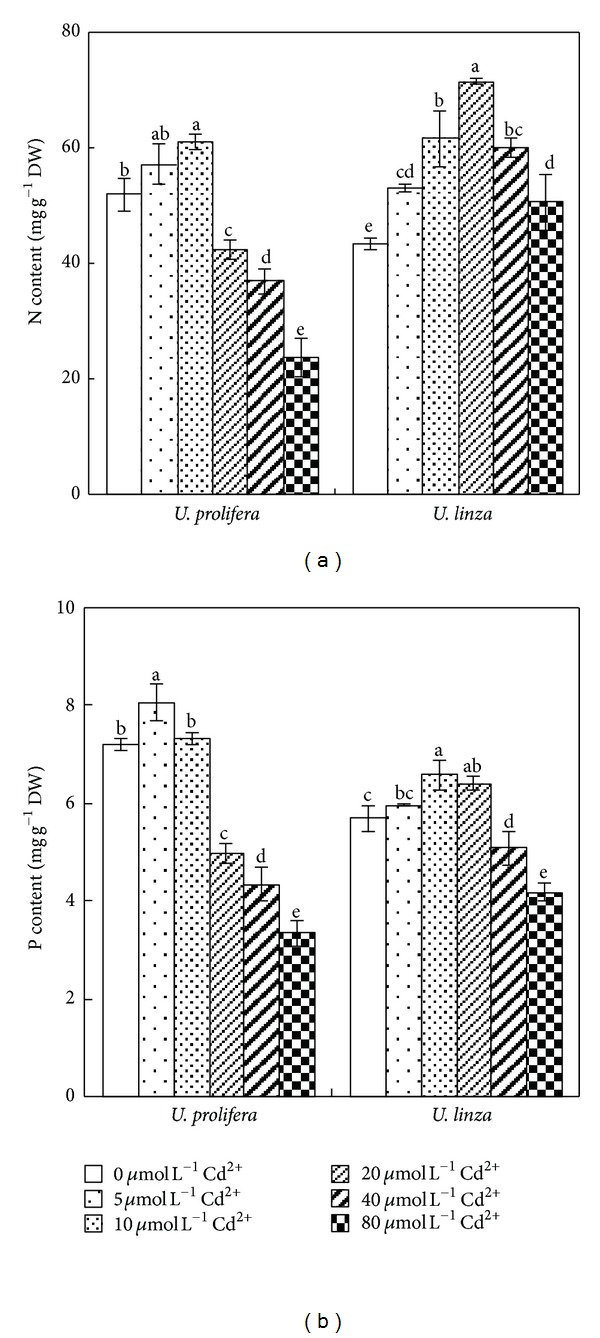
Effects of different concentrations of Cd^2+^ (0, 5, 10, 20, 40, 80 *μ*mol L^−1^) on contents of N (a) and P (b) of *U. prolifera* and *U. linza. *

Contents of N and P in both *Ulva* species showed a declining trend after an initial increase. The highest N content was recorded at 10 *μ*mol L^−1^ Cd^2+^ in *U. prolifera* and at 20 *μ*mol L^−1^ Cd^2+^ in *U. linza*. N contents in *U. linza* in all Cd^2+^ treatments were higher than those of control; however, in *U. prolifera*, N contents at 20, 40, or 80 *μ*mol L^−1^ Cd^2+^ were significantly decreased compared to the control (Figure 6(a)). 

*U. prolifera* had the highest P concentration at 5 *μ*mol L^−1^ Cd^2+^; but the highest P concentration was observed when *U. linza* was treated by 10 *μ*mol L^−1^ Cd^2+^. The P contents decreased 31, 40, and 54% at 20, 40, and 80 *μ*mol L^−1^ Cd^2+^ in *U. prolifera,* respectively. Compared to the control, the P concentration of *U. linza* at 20 *μ*mol L^−1^ Cd^2+^ increased significantly, and then decreased by 11 and 27% under 40, and 80 *μ*mol L^−1^ Cd^2+^, respectively (Figure 6(b)). 

### 3.6. Effect of Cadmium Stress on Inorganic Elements of *U. prolifera* and *U. linza*

The Na^+^ content of *U. prolifera* grown at 5 or 10 *μ*mol L^−1^ Cd^2+^ was not significantly different from the control, and it increased by 42, 67, and 83% at 20, 40, and 80 *μ*mol L^−1^ Cd^2+^, respectively. However, in *U. linza*, 5, 10, 20, and 40 *μ*mol L^−1^ Cd^2+^ had no significant influence on Na^+^ content, and 80 *μ*mol L^−1^ Cd^2+^ increased Na^+^ content by 36% (Table 1). The K^+^ content of *U. prolifera* grown at 5 or 10 *μ*mol L^−1^ Cd^2+^ remained unaffected compared to the control; it decreased significantly by 41, 45, and 62% at 20, 40, and 80 *μ*mol L^−1^ Cd^2+^, respectively. In *U. linza*, 5, 10, and 20 *μ*mol L^−1^ Cd^2+^ had no significant influence on K^+^ content, whereas 40 and 80 *μ*mol L^−1^ Cd^2+^ decreased K^+^ content by 34 and 50%, respectively (Table 1). The Ca^2+^ content of *U. prolifera* grown at 5, 10, 20, or 40 *μ*mol L^−1^ Cd^2+^ remained unaffected, but increased significantly (24%) at 80 *μ*mol L^−1^ Cd^2+^. However, in *U. linza*, 5 and 10 *μ*mol L^−1^ Cd^2+^ had no significant influence on Ca^2+^ contents, whereas 20, 40, and 80 *μ*mol L^−1^ Cd^2+^ increased Ca^2+^ content by 22, 39, and 50%, respectively (Table 1). The Mg^2+^ content of *U. prolifera* grown at 5, 10, 20, 40 or 80 *μ*mol L^−1^ Cd^2+^ remained unaffected. With increasing Cd^2+^ concentrations, Mg^2+^ contents of *U. linza* showed an increasing trend after an initial decline (Table 1). The Cl^−^ contents appeared to have a declining trend with increasing Cd^2+^ concentration similarly to Mg concentrations. However, no obvious difference in Cl^−^ contents among all Cd^2+^ treatments was noted in the two *Ulva* species (Table 1). Nitrate content in *U. prolifera* showed an uptrend with increasing Cd^2+^ concentration; however, with increasing Cd^2+^ concentrations, nitrate content of *U. linza* showed a decline trend after an initial increase. We also found that nitrate contents of *U. linza* were much more than those of *U. prolifera *under all treatments except for 80 *μ*mol L^−1^ Cd^2+^ treatment (Table 1). 

The K^+^/Na^+^ and Ca^2+^/Na^+^ ratios in *U. prolifera* were not influenced by 5 and 10 *μ*mol L^−1^ Cd^2+^, but they showed declining trends at 20, 40, and 80 *μ*mol L^−1^ Cd^2+^ (Table 1). In *U. linza*, 5 and 10 *μ*mol L^−1^ Cd^2+^ had no significant influence on the K^+^/Na^+^ ratio, whereas 20, 40, and 80 *μ*mol L^−1^ Cd^2+^ decreased that ratio by 6, 45, and 64%, respectively. However, in *U. prolifera*, 20, 40, and 80 *μ*mol L^−1^ Cd^2+^ decreased the K^+^/Na^+^ ratio by 55, 65, and 78%. No Cd^2+^ treatment significantly changed the Ca^2+^/Na^+^ ratio in *U. linza*. 

### 3.7. Effect of Cadmium Stress on Organic Solutes in *U. prolifera* and *U. linza*

With increasing Cd^2+^ concentration, soluble sugar (SS) content appeared to have an ascending trend after an initial decline in both *Ulva* species. In *U. prolifera*, 40 *μ*mol L^−1^ Cd^2+^ did not change the SS content, and 80 *μ*mol L^−1^ Cd^2+^ increased SS concentration by 27% compared to the control. However, in *U. linza*, 40 and 80 *μ*mol L^−1^ Cd^2+^ increased SS content by 40 and 90%, respectively (Table 2). In *U. prolifera *and *U. linza*, 5 *μ*mol L^−1^ Cd^2+^ significantly increased free amino acid (FAA) content by 25 and 16%, respectively. However, 10 *μ*mol L^−1^ Cd^2+^ had no obvious change on FAA contents of the two *Ulva* species. Treatments with 20, 40, and 80 *μ*mol L^−1^ Cd^2+^ significantly decreased FAA content by 52, 79, and 87% in *U. prolifera* and by 2, 25, and 43% in *U. linza *(Table 2). Proline (PRO) content was greatly enhanced by Cd^2+^ treatments in both *Ulva* species. At 5, 10, 20, 40, and 80 *μ*mol L^−1^ Cd^2+^, PRO content was increased 154, 431, 715, 1031, and 1069%, respectively, in *U. prolifera*; and increased 147, 420, 726, 1040, and 1147%, respectively, in* U. linza* (Table 2). Organic acid (OA) content in* U. prolifera* was not affected at 5, 10 and 20 *μ*mol L^−1^ Cd^2+^, and OA concentration in *U. linza* was not affected at 5, 10, 20, and 40* μ*mol* *L^−1^ Cd^2+^. Treatments with 40 and 80 *μ*mol L^−1^ Cd^2+^ decreased OA content by 29 and 47%, respectively, in *U. prolifera*, whereas in *U. linza* only 80 *μ*mol L^−1^ Cd^2+^ decreased OA content by 27% (Table 2). The soluble protein (SP) content in the two *Ulva* species was not affected at 5, 10 and 20* μ*mol* *L^−1^ Cd^2+^ and was decreased at 40 and 80* μ*mol* *L^−1^ Cd^2+^. Treatments with 40 and 80 *μ*mol L^−1^ Cd^2+^ significantly decreased SP content by, respectively, 16 and 42% in *U. prolifera* and by 8 and 25% in *U. linza *(Table 2). 

### 3.8. Correlation Analysis between RGR and Other Physiological and Biochemical Indexes under Cadmium Stress

Correlation analysis indicated that RGR of both *Ulva* species was insignificantly related to contents of Chl, Car, Na^+^ and Mg^2+^, and the Ca^2+^/Na^+^ ratio. In contrast, RGR was highly negative correlated with the contents of Cd^2+^, Ca^2+^, SS, and PRO, and highly positive correlated with the contents of N, P, K, Cl, FAA, OA and SP, K^+^/Na^+^ ratio, OAA, Fv/Fm, and Yield (Table 3).

## 4. Discussion

Plant growth can be suppressed by Cd [7, 17]. It was reported that *Ulva lactuca* was sensitive to cadmium, as obviously shown by growth reduction and lethal effects at 40 *μ*mol L^−1^ Cd^2+^ within 6 days [27]. In the study presented here, *U. prolifera* and *U. linza*, the dominant free-floating *Ulva *species of green tide bloom in the Yellow Sea of China [28], showed sensitivity to Cd^2+^ (reduction in RGR, Fv/Fm, and Yield). Furthermore, this reduction was found to be more pronounced in *U. prolifera *than *U. linza*. After 7 d, *U. prolifera* died at 120 *μ*mol L^−1^ Cd^2+^, whereas *U. linza* was still alive (Figures 1 and 4). This result indicated that *U. linza *had better adaptation to Cd^2+^ toxicity than *U. prolifera*.

It is known that marine macroalgae can concentrate heavy metals to a large extent [2, 29]. In this study, Cd accumulation in *U. prolifera *and *U. linza* increased significantly in response to increased Cd^2+^ concentrations. However, *U. prolifera* accumulated more Cd than *U. linza* (Figure 3). In general, plant accumulation of a given metal is a function of uptake capacity and intracellular binding sites [30]. The cell walls of plant cells contain proteins and different carbohydrates that can bind metal ions. After the binding sites in the cell wall become saturated, intracellular Cd accumulation mediated by metabolic processes may lead to cell toxicity [31]. 

*Ulva* species are widely distributed in the coastal intertidal zones where had full change on salinity level. Thus, many *Ulva* species have strong OAA to cope with variable and heterogeneous environments. Similarly to a number of other stresses, heavy metal toxicity can decrease cell water content and lower the cell water potential (*ψ*_*w*_) through increased net concentrations of solutes (osmotic adjustment), which is a common response to water stress and an important mechanism for maintaining cell water content and, thus, turgor [18, 32]. In our experiments, OAA of *U. linza *had positive values in the treatments with 5, 10, or 20 *μ*mol L^−1^ Cd^2+^, whereas *U. prolifera* had positive OAA only at 5 and 10 *μ*mol L^−1^ Cd^2+^ (Figure 2). When OAA values in *Ulva* were positive, that is, OAA contributed to maintaining turgor, *Ulva* could continue growing, and RGR was positive. However, when OAA in *Ulva* was negative resulting in turgor loss, the growth was stopped, and RGR was negative. Correlation analysis also showed that RGR was positively related to OAA, suggesting that OAA played an important role in maintaining algal growth. Also, good osmotic adjustment enabled plants to maintain high photosynthetic activity (Figure 5).

Cadmium is a nonessential element for plant growth, and it inhibits uptake and transport of many macro- and micronutrients, inducing nutrient deficiency [7, 17]. Contradictory data can be found in the literature on the effects exerted by Cd^2+^ on terrestrial plant. Cadmium was reported to reduce uptake of N, P, K, Ca, Mg, Fe, Zn, Cu, Mn, Ni, and Na in many crop plants [33], whereas other authors found reduced K uptake but unchanged P uptake or even an increase in K content of several crop varieties under Cd^2+^ stress [34, 35]. Obata and Umebayashi [36] reported that Cd^2+^ treatment increased Cu content in the roots of pea, rice, and maize, but unchanged Cu content in cucumber and pumpkin plants. With Cd^2+^ stress, Maksimović et al. [37] observed a reduction in the maize root influx and root-shoot transport of Cu, Zn, and Mn, a reduction in the root-shoot transport of Fe, but an increase in Fe influx and Ca and Mg transport. In this study, the response of total N and P concentrations in tissues of the two *Ulva* species to Cd^2+^ treatments was positively related to their Cd resistance. We found that the treatment with low concentration of Cd^2+^ enhanced N and P contents, but high concentrations of Cd^2+^ (≥20 *μ*mol L^−1^) decreased N and P contents in both *Ulva* species. The maintenance of total N and total P was more pronounced in less Cd-sensitive *U. linza* than Cd-sensitive *U. prolifera *(Figure 6). This suggests that the maintenance of a normal level of total N content upon challenge with Cd is likely to be a feature in relative Cd-resistant marine macroalga, similarly to terrestrial plants [38]. In *Ulva*, we found that the contents of K^+^, Ca^2+^, and Cl^−^ were related to RGR, especially K^+^reduction caused *Ulva* growth reduction significantly (Table 1). Thus, the K^+^/Na^+^ ratio in both *Ulva* species decreased significantly with increasing Cd^2+^ treatment concentrations, and Cd^2+^-sensitive *U. prolifera *showed a greater K^+^/Na^+^ decline than Cd^2+^-sensitive *U. linza* (Table 1). 

We measured a decline in soluble sugar (SS) concentration at low Cd^2+^ treatment concentrations and an increase at high Cd^2+^ concentrations in both *Ulva* species. Moreover, the SS increase of *U. linza* is more marked than that of *U. prolifera*. In other studies, the decline in SS concentration corresponded with the photosynthetic inhibition or stimulation of respiration rate, affecting carbon metabolism and leading to production of other osmotica [39]. The accumulating soluble sugars in plants growing in presence of Cd^2+^ could provide an adaptive mechanism via maintaining a favorable osmotic potential under adverse conditions of Cd^2+^ toxicity [40]. 

Soluble protein (SP) content in organisms is an important indicator of metabolic changes and responds to a wide variety of stresses [41]. In this work, SP contents in *U. prolifera *and *U. linza* declined with increasing Cd^2+^ treatment concentrations. Free amino acid (FAA) contents in both *Ulva *species first increased and then declined, with such a decline more pronounced in *U. prolifera* than in *U. linza*. The decreased protein content together with the increased free amino acid content suggest that the protein synthesizing machinery was impaired due to the Cd^2+^ effect [42]. 

PRO accumulation in plant tissues in response to a number of stresses, including drought, salinity, extreme temperatures, ultraviolet radiation, or heavy metals, is well documented [43]. In this study, even though PRO content was increased in Cd^2+^-treated *Ulva*, its absolute amount was relatively low. Under assumed localization of inorganic ions in the vacuole and organic solutes in the cytoplasm, FAA and PRO may be mainly in the cytoplasm, accounting for about 5%–10% volume in mature cells [44]. A small amount of FAA and PRO accumulating in the cytoplasm can increase concentration significantly and play an important role in balancing vacuolar osmotic potential [44]. It has often been suggested that PRO accumulation may contribute to osmotic adjustment at the cellular level [39]. In addition, PRO as a compatible solute may protect enzymes from dehydration and inactivation [18]. 

In conclusion, exposing *U. prolifera *and *U. linza* to different concentrations of Cd^2+^ resulted in the changes in growth, pigment content, chlorophyll fluorescence parameters, Cd accumulation, OAA, and concentration of N, P, main inorganic ions, and organic solutes. These changes make *U. linza *better adapted to withstanding Cd^2+^ stress in comparison with *U. prolifera*. Our results highlight the role of osmotic adjustment in *Ulva* during Cd^2+^ stress as an important mechanism enabling *Ulva* to maintain photosynthetic activity and, thus, growth under Cd^2+^ stress. 

## Figures and Tables

**Table 1:** Effects of different concentrations of Cd^2+^ (0, 5, 10, 30, 40, and 80 *μ*mol L^−1^) on inorganic ion content (mmol g^−1^ DW), K^+^/Na^+^ and Ca^2+^/Na^+^ of *U. prolifera* and* U. linza. *

	Cd^2+^ treatment	Na^+^	K^+^	Ca^2+^	Mg^2+^	Cl^−^	NO_3_^−^	K^+^/Na^+^	Ca^2+^/Na^+^
	*μ*mol L^−1^	mmol g^−1^ DW	mmol g^−1^ DW	mmol g^−1^ DW	mmol g^−1^ DW	mmol g^−1^ DW	mmol g^−1^ DW		
*U. prolifera *	0	0.12 ± 0.01 c	0.64 ± 0.04 a	0.20 ± 0.02 b	0.82 ± 0.04 a	0.15 ± 0.01 a	0.34 × 10^−3^ ± 0.03 × 10^−3^ c	5.01 ± 0.12 a	1.70 ± 0.08 a
	5	0.13 ± 0.02 c	0.62 ± 0.05 a	0.23 ± 0.01 ab	0.78 ± 0.05 a	0.11 ± 0.01 b	0.49 × 10^−3^ ± 0.06 × 10^−3^ c	4.86 ± 0.21 a	1.66 ± 0.07 a
	10	0.12 ± 0.01 c	0.63 ± 0.04 a	0.23 ± 0.02 ab	0.76 ± 0.05 a	0.10 ± 0.01 b	0.78 × 10^−3^ ± 0.06 × 10^−3^ b	5.10 ± 0.14 a	1.84 ± 0.12 a
	20	0.17 ± 0.02 b	0.38 ± 0.03 b	0.23 ± 0.02 ab	0.75 ± 0.04 a	0.09 ± 0.01 b	1.41 × 10^−3^ ± 0.08 × 10^−3^ a	2.26 ± 0.15 b	1.39 ± 0.10 b
	40	0.20 ± 0.02 ab	0.35 ± 0.03 b	0.25 ± 0.02 ab	0.79 ± 0.04 a	0.10 ± 0.01 b	1.40 × 10^−3^ ± 0.11 × 10^−3^ a	1.74 ± 0.11 c	1.24 ± 0.08 bc
	80	0.22 ± 0.01 a	0.24 ± 0.02 c	0.26 ± 0.02 a	0.73 ± 0.05 a	0.10 ± 0.01 b	1.43 × 10^−3^ ± 0.04 × 10^−3^ a	1.12 ± 0.08 d	1.14 ± 0.07 c

*U. linza *	0	0.25 ± 0.02 b	0.74 ± 0.04 a	0.18 ± 0.02 c	0.78 ± 0.04 a	0.16 ± 0.01 a	0.86 × 10^−3^ ± 0.08 × 10^−3^ d	3.02 ± 0.15 a	0.72 ± 0.09 a
	5	0.24 ± 0.03 b	0.74 ± 0.04 a	0.17 ± 0.02 c	0.75 ± 0.03 ab	0.12 ± 0.01 b	1.21 × 10^−3^ ± 0.10 × 10^−3^ c	3.10 ± 0.23 a	0.73 ± 0.08 a
	10	0.24 ± 0.02 b	0.73 ± 0.04 a	0.18 ± 0.01 c	0.72 ± 0.04 ab	0.10 ± 0.02 b	1.89 × 10^−3^ ± 0.07 × 10^−3^ a	3.04 ± 0.12 a	0.75 ± 0.06 a
	20	0.24 ± 0.01 b	0.68 ± 0.03 a	0.22 ± 0.02 b	0.64 ± 0.03 b	0.10 ± 0.01 b	2.07 × 10^−3^ ± 0.12 × 10^−3^ a	2.85 ± 0.11 b	0.83 ± 0.07 a
	40	0.26 ± 0.02 b	0.49 ± 0.03 c	0.25 ± 0.02 ab	0.68 ± 0.03 b	0.11 ± 0.01 b	1.65 × 10^−3^ ± 0.05 × 10^−3^ b	1.67 ± 0.07 c	0.88 ± 0.08 a
	80	0.34 ± 0.02 a	0.37 ± 0.02 d	0.27 ± 0.02 a	0.72 ± 0.04 ab	0.12 ± 0.02 b	1.12 × 10^−3^ ± 0.11 × 10^−3^ c	1.10 ± 0.05 d	0.75 ± 0.06 a

The data in the same column are statistically different if labeled with different letters according to Duncan's multiple range test (*P* ≤ 0.05).

**Table 2:** Effects of different concentration of Cd^2+^ (0, 5, 10, 30, 40, and 80 *μ*mol L^−1^) on organic solute content of *U. prolifera* and *U. linza*.

	Cd^2+^ treatment	SS	FAA	PRO	OA	SP
	*μ*mol L^−1^	mmol g^−1^ DW	mmol g^−1^ DW	mmol g^−1^ DW	mmol g^−1^ DW	mg g^−1^ DW
*U. prolifera *	0	0.15 ± 0.02 b	1.03 ± 0.05 b	0.13 × 10^−3^ ± 0.02 × 10^−3^ e	0.17 ± 0.01 a	42.15 ± 2.33 a
	5	0.15 ± 0.02 b	1.29 ± 0.12 a	0.33 × 10^−3^ ± 0.02 × 10^−3^ d	0.17 ± 0.01 a	41.38 ± 2.76 a
	10	0.12 ± 0.01 bc	1.10 ± 0.04 ab	0.69 × 10^−3^ ± 0.04 × 10^−3^ c	0.19 ± 0.02 a	40.45 ± 1.86 a
	20	0.10 ± 0.01 c	0.49 ± 0.11 c	1.06 × 10^−3^ ± 0.07 × 10^−3^ b	0.16 ± 0.02 a	38.39 ± 2.75 ab
	40	0.14 ± 0.01 b	0.22 ± 0.05 d	1.47 × 10^−3^ ± 0.09 × 10^−3^ a	0.12 ±0.01 b	35.53 ± 2.63 b
	80	0.19 ± 0.01 a	0.13 ± 0.06 d	1.52 × 10^−3^ ± 0.12 × 10^−3^ a	0.09 ± 0.01 c	24.35 ± 1.88 c

*U. linza *	0	0.10 ± 0.01 cd	1.23 ± 0.03 b	0.15 × 10^−3^ ± 0.05 × 10^−3^ f	0.11 ± 0.02 a	39.27 ± 1.22 a
	5	0.10 ± 0.01 cd	1.43 ± 0.09 a	0.37 × 10^−3^ ± 0.02 × 10^−3^ e	0.12 ± 0.01 a	38.89 ± 2.37 ab
	10	0.07 ± 0.01 d	1.21 ± 0.10 b	0.78 × 10^−3^ ± 0.03 × 10^−3^ d	0.13 ± 0.01 a	38.52 ± 2.67 ab
	20	0.10 ± 0.01 c	1.20 ± 0.06 b	1.24 × 10^−3^ ± 0.08 × 10^−3^ c	0.14 ± 0.02 a	37.13 ± 1.89 ab
	40	0.14 ± 0.02 b	0.97 ± 0.06 c	1.71 × 10^−3^ ± 0.07 × 10^−3^ b	0.12 ± 0.01 a	35.95 ± 2.41 b
	80	0.19 ± 0.01 a	0.76 ± 0.08 d	1.87 × 10^−3^ ± 0.15 × 10^−3^ a	0.08 ± 0.01 b	29.34 ± 1.87 c

Different letters in the same column indicate statistical difference according to Duncan's multiple range test (*P* ≤ 0.05). “SS, FAA, PRO, OA, and SP” in the table indicate the content of soluble sugar, free amino acid, proline, organic acid, and soluble protein, respectively.

**Table 3:** Correlation coefficients between RGR and other indices for *U.  prolifera* and *U.  linza*.

Index	Correlation coefficient
Chl content	0.072
Car content	0.198
Fv/Fm	0.830**
Yield	0.858**
Cd^2+^ content	−0.899**
N content	0.561**
P content	0.687**
OAA	0.766**
Na^+^ content	−0.138
K^+^ content	0.881**
Ca^2+^ content	−0.677**
Mg^2+^ content	0.060
Cl^−^ content	0.444**
K^+^/Na^+^	0.627**
Ca^2+^/Na^+^	−0.079
SS content	−0.617**
FAA content	0.828**
PRO content	−0.841**
OA content	0.731**
SP content	0.752**

*Significant at 5% level, **significant at 1% level (two-tailed, *n* = 18).
